# Enhancing High‐Humidity Stability of CsPbI_3_ Perovskite Solar Cells Through Strong Bidentate Ligand Coordination

**DOI:** 10.1002/smll.73854

**Published:** 2026-05-20

**Authors:** Karthikeyan Embrose, Thangaraji Vasudevan, Lung‐Chien Chen

**Affiliations:** ^1^ National Taipei University of Technology Taipei Taiwan

**Keywords:** additive engineering, defects passivation, high durability and efficiency, inorganic perovskite, solar cells

## Abstract

The stability of inorganic CsPbI_3_ perovskite solar cells (IPSCs) has been greatly limited by their accelerated degradation in a high‐humidity environment. In this investigation, we design a defect‐passivation approach using monodentate p‐toluenesulfonyl hydrazide (TSH) and a bidentate ligand, 2‐amino‐6‐methoxybenzothiazole (AMBT), which boosts the humidity resistance of CsPbI_3_ perovskite under 80% relative humidity (RH). Based on density‐functional‐theory calculations, it has been found that AMBT has a significantly higher binding energy of −1.91 eV with respect to CsPbI_3_ than that of TSH with a binding energy of −0.98 eV, consistent with their extremely stable Pb‐N and Pb─O coordination bonds, ascertained by XPS and FTIR spectroscopy. Surface modification of AMBT effectively promotes crystal perfection, decreases trap state, and extends carrier lifetime, yielding a maximum power conversion efficiency (PCE) of 18.52% with a high fill factor of 84.35%. Unencapsulated device structures with 80% RH maintain their β‐phase structure for over 60 min, and corresponding IPSCs maintain 40% of their initial efficiency over 10 days, being among the highest humidity‐resistance strengths for CsPbI_3_ perovskite‐based devices. Our results provide a powerful strategy for developing moisture‐independent CsPbI_3_ perovskite solar cells.

## Graphical abstract

Inorganic CsPbI_3_ perovskite solar cells suffer from rapid phase degradation under high humidity. We show that bidentate coordination using 2‐amino‐6‐methoxybenzothiazole (AMBT) provides significantly stronger defect passivation than monodentate p‐toluenesulfonyl hydrazide, achieving a binding energy of −1.91 eV with the perovskite surface. The AMBT‐passivated unencapsulated devices deliver 18.52% efficiency and retain 40% of initial efficiency after 10 days at 80% relative humidity.

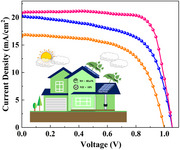

## 1 Introduction

OIHP (Organic–inorganic hybrid perovskite solar cells) are being accepted as potential options for commercial development, mostly due to their superior photovoltaic (PV) property, easy processing techniques, and relatively low costs [1, 2, 3]. The certified PCE (power conversion efficiencies) values of OIHPs are around 27.0% [4]. Despite these major developments, OIHPs also have natural limitations that cause serious challenges, such as decomposition, thermodynamics, and volatilization owing to evaporation of organic compounds at high temperatures [5], and sensitivity to oxygen and humidity. These problems can, in principle, be solved by replacing organic compounds with inorganic cations [6, 7, 8, 9, 10].

The theoretical calculations reveal that the initial highest efficiency for inorganic perovskite solar cells (IPSCs), CsPbI_3_, can reach about 28% [11]. This value corresponds to that of the most advanced silicon solar cell. One of the most interesting application areas of IPSCs, especially for their use in tandems, is their potential for bandgap‐engineering with silicon without needing major adjustments of the halide ratios, which help to suppress known issues with halo‐segregation upon prolonged illumination [12, 13, 14, 15]. Much effort has been directed toward enhancing IPSC in various ways, including advancements in their structural architectures [16, 17], compositional engineering [18, 19, 20, 21, 22], crystallinity [23, 24, 25, 26], and interfaces [27, 28, 29, 30, 31]. These developments enabled a tremendous increase in their efficiency from 2.5% in 2015 [32] to 21.7% in recent studies [33].

CsPbI_3_ perovskite films prepared through solution processing typically are polycrystalline in nature, wherein the fast crystallization process prompts a very high density of structural defects [33, 34]. These defects tend to behave as nonradiative recombination centers and trap states, which impede efficient charge carrier transport. Consequently, the metastable black polymorphs (α/β/γ‐phase) tend to easily transform to the optically inert yellow δ‐phase, along with the collapse of the corner‐sharing framework of [PbI_6_]^4−^ octahedra. This structural degrading, in turn, further accelerates phase instability and eventually decomposes the perovskite lattice into smaller fragments [35, 36]. To combat these issues, several strategies were developed involving surface modification [37, 38, 39] and the fabrication of gradient heterojunctions [40] with the aim of reducing the deficit in open‐circuit voltage, V_OC_, and enhancing phase stability. For example, Mali et al. used (Zn(C_6_F_5_)_2_) to reconstruct the surface of thin perovskite films through graded heterojunction formation that tends to simultaneously enhance stability and PCE [41]. In addition, the construction of phase heterojunctions within CsPbI_3_‐based inverted PSCs was also reported to yield a high efficiency of 21.59% while maintaining good V_OC_ and long‐term operational stability. Beyond surface and interface engineering, bulk doping by organic molecules, such as 2,6‐diaminopyridine (2,6‐DAPY) and versatile molten salts like DMAAc, has also prompted further improvement in both efficiency and phase stability [42, 43, 44, 45]. In those ways, IPSCs have achieved a record efficiency of 21.80% [46] with significantly reduced defect density and a very minimized V_OC_ deficit with a bandgap of 1.67 eV.

The low formation energy of halogen vacancies makes them the dominant ones in CsPbI_3_, and accordingly, Lewis bases eliminate such defects by coordinating with undercoordinated Pb^2+^ /Cs^+^ ions [47, 48, 49, 50]. Wu et al. used 2‐(4‐aminophenyl) ethylamine to stabilize cubic CsPbI_3_ and achieved 19.5% efficiency with excellent durability [51], whereas monodentate passivation is fragile under solvents or heat [52]. Bidentate and multidentate ligands with stronger coordination provide more robust defect binding and stability [56]; for instance, Xu et al. demonstrated that 2‐mercapto‐1‐methylimidazole (MMI) could reduce defects and boost the PCE to 20.6% [53]. Such progress confirms that multidentate ligand design is one critical path toward highly efficient and stable all‐inorganic PSCs.

Accordingly, in the current work, we have studied monodentate p‐toluenesulfonyl hydrazide (TSH) and bidentate 2‐amino‐6‐methoxybenzothiazole (AMBT) as defect‐suppressing additives in CsPbI_3_ films. TSH coordinates with undercoordinated Pb^2+^/Cs^+^ via its ‐NHNH_2_ group, while AMBT coordinates through a dual site: the benzothiazole ring and the amino group. The stronger bidentate interaction of AMBT increases Pb^2+^/Cs^+^ binding and defect formation energies, hence giving rise to superior charge extraction, stability, and device performance. The highest PCE of 18.52% (0.04 cm^2^) was obtained from AMBT‐passivated CsPbI_3_ solar cells. Besides this, we for the first time demonstrate that an uncapped AMBT‐passivated CsPbI_3_ film can maintain its β‐phase over 60 min under ambient conditions of 25 °C and 80% relative humidity‐the longest ever reported to the best of our knowledge. More importantly, 40% of the initial PCE is preserved after 10 days under the same conditions, revealing significantly improved operational stability.

## 2 Results and Discussion

**FIGURE 1:**
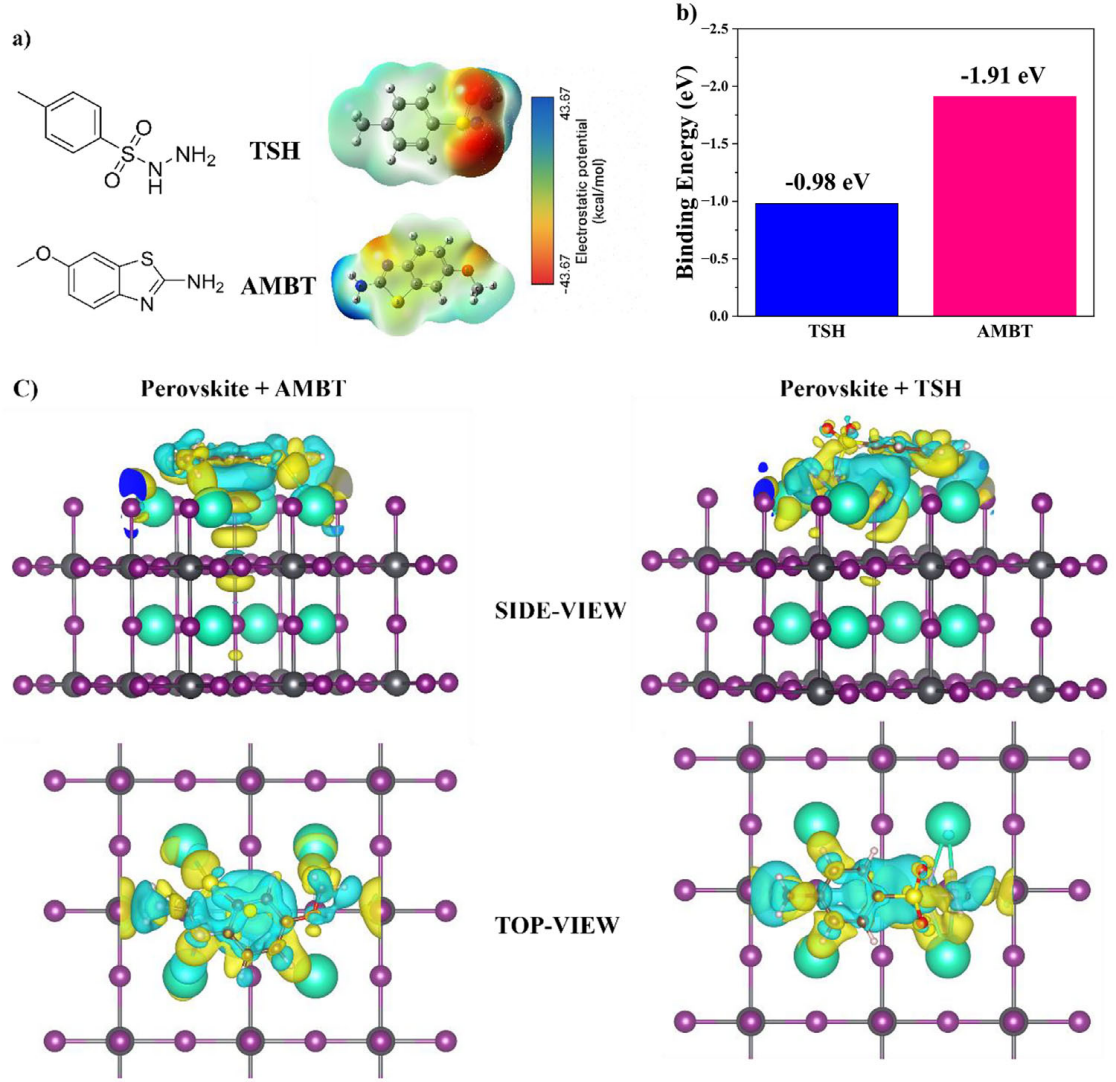
Theoretical analysis of perovskite defects and additive interactions. (a) Chemical structures and electrostatic potential (ESP) maps of TSH and AMBT. (b) Binding energies between TSH/AMBT and undercoordinated Pb^2+^ or Cs^+^ sites. (c) Side and top views of charge‐density differences at the perovskite TSH/AMBT interface, where cyan indicates electron depletion and yellow indicates electron accumulation.

Density functional theory (DFT) calculations were conducted to explain the interaction of CsPbI_3_ surfaces with the organic passivators 2‐amino‐6‐methoxybenzothiazole (AMBT) and p‐toluenesulfonyl hydrazide (TSH). Their molecular structures and ESP maps (Figure 1a) show distinctly different charge distributions. TSH has only one highly electron‐rich region (−43.67 kcal mol^−1^), from the sulfonyl oxygen or hydrazide nitrogen atoms, which are suitable for Lewis's base interactions localized at undercoordinated Pb^2+^ or Cs^+^ sites. In contrast, a rather delocalized electron‐rich environment (−38.70 kcal mol^−1^) is evident in AMBT, encompassing the N, O, and S heteroatoms. This creates a distributed molecular dipole that would favor stable surface orientation and more uniform interfacial contact. Such a spatially extended donor site is expected to enhance surface coverage despite the lower peak ESP intensity. The relevant structural models of the perovskite additive are given in Figure S1.

Quantification of these interactions by calculating adsorption energies and charge‐density difference (CDD) profiles is shown in Figure 1b,c and Table S1. AMBT was found to exhibit significantly stronger binding to the CsPbI_3_ surface compared to that of TSH, as indicated by adsorption energies of −1.91 versus −0.98 eV, respectively. The high binding strength of AMBT arises from its bidentate coordination, where the amino and methoxy groups interact simultaneously with surface Pb and I atoms. In contrast, TSH binds in a primarily monodentate fashion through either the sulfonyl or hydrazide moiety, leading to weaker surface affinity.

These trends are further corroborated by the CDD isosurfaces. Indeed, in the case of AMBT, significant charge accumulation around the amino N and methoxy O atoms and depletion around the neighboring Pb and I atoms evidence an efficient donor‐acceptor interaction along with partial covalent Pb─N and Pb─O bonding. The electron redistribution extends into deeper layers of Pb‐I, reflecting strong orbital hybridization. On the contrary, TSH exhibits a charge rearrangement which is more localized and significantly weaker, confined to the immediate contact region, being thus consistent with prevailing physisorptive interactions due to hydrogen bonding and van der Waals forces. The top‐view CDD map confirms indeed its much less effective electronic coupling with the perovskite surface.

**FIGURE 2:**
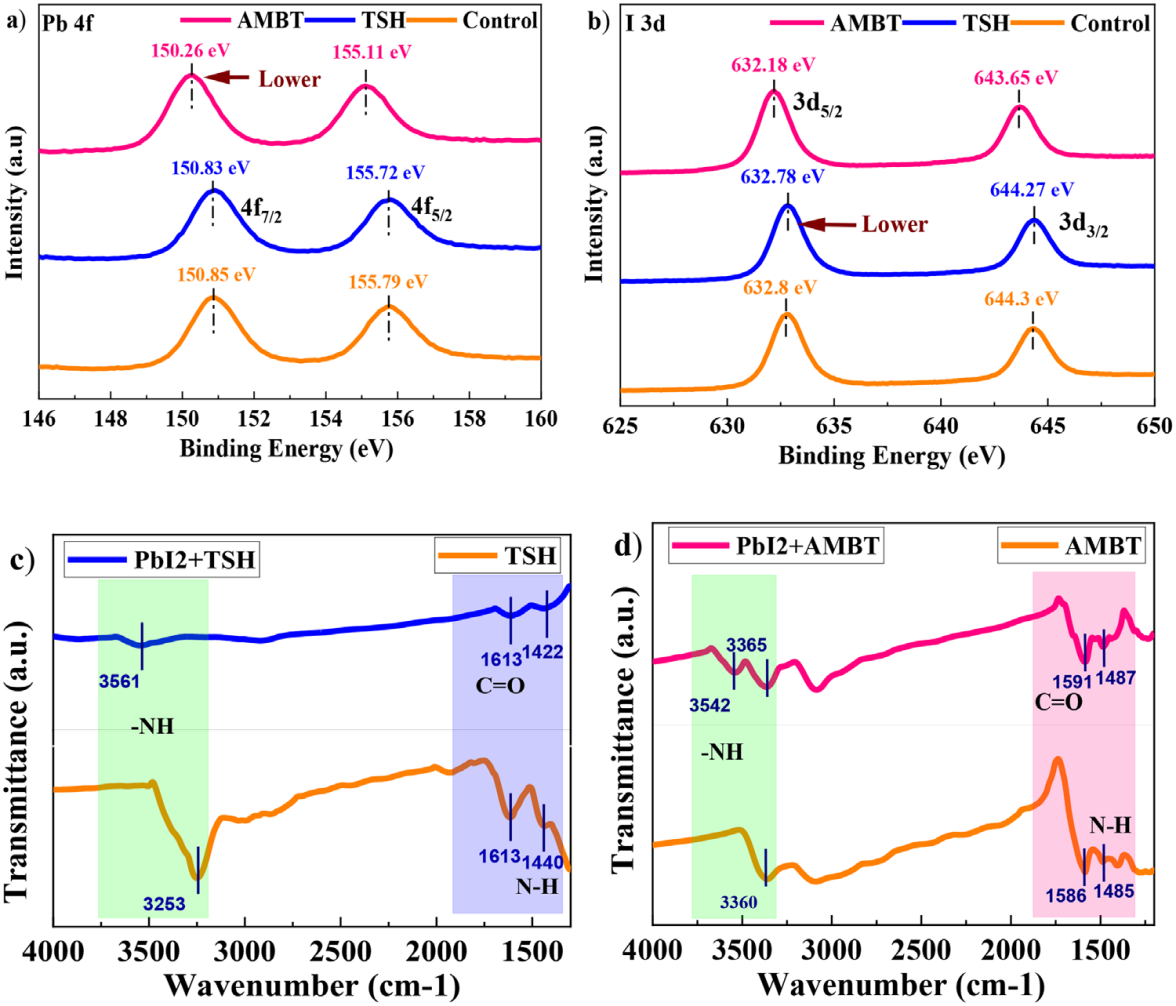
(a) Pb 4f XPS spectra, and (b) I 3d XPS spectra for control and Additive‐modified CsPbI_3_ films. (c) FTIR spectra of pure TSH and TSH‐PbI_2_ mixtures. (d) FTIR spectra of pure AMBT and AMBT‐PbI_2_ mixtures.

X‐ray photoelectron spectroscopy (XPS) was carried out to investigate the interaction strength between additive molecules and the CsPbI_3_ perovskite surface. As illustrated in Figure 2a,b, for the pristine film, the Pb 4f5/2 and 4f7/2 peaks are located at 155.79 and 150.85 eV, while the I 3d3/2, and I 3d5/2 peaks are centered at 644.30, and 632.80 eV, respectively. After the addition of AMBT, these peaks apparently shift toward lower binding energy, with peaks positioned at 155.11, 150.26, 643.65, and 632.18 eV, respectively, demonstrating strong electronic interactions between AMBT and the perovskite lattice. In contrast, the TSH‐modified film only presents a small peak shift, indicating weaker interactions. Meanwhile, the N 1s, O 1s and Cs 3d, peaks of both the AMBT and TSH modified films slightly shift toward higher binding energies (Figure S2a–c), which also confirms the chemical bonding across the interface. These demonstrate that AMBT and TSH coordinate with Pb^2+^ via C═O and N─H groups and form hydrogen bonds with I^−^ through the ‐NH_2_ functionalities. Moreover, the larger shifts of the peaks for AMBT demonstrate stronger coordination due to the electron‐donating methoxy group and p‐π conjugation of the benzothiazole ring. Meanwhile, the presence of the hydrazide group in TSH also affords effective passivation. Overall, the obtained XPS results agree well with DFT predictions, which confirm that AMBT and TSH have a passivation effect on CsPbI_3_. For investigating the coordination interaction between additive molecules and undercoordinated Pb^2+^ ions, PbI_2_ and corresponding passivators were dissolved in DMSO and characterized using Fourier‐transform infrared (FTIR) spectroscopy (Figure 2c,d). In TSH, ‐NH_2_ stretching vibration from 3253 to 3561cm^−1^ and N─H bending at 1485 cm^−1^ shifted slightly to higher wavenumbers after adding PbI_2_, which resulted from the Pb─N coordination. In the AMBT sample, two ‐NH_2_ stretching peaks at 3365 and 3542 cm^−1^ also shifted to higher wavenumbers after incorporating PbI_2_, which resulted from chelation of the two N─H groups to Pb^2+^, Besides, the C═O and N─H modes at 1586 and 1485 cm^−1^ shifted similarly, which further confirms the formation of a Pb─O bond and a Pb─N bond.

Collectively, the XPS and FTIR data provide complementary evidence that AMBT forms stronger Pb─O and Pb─N coordination bonds with undercoordinated Pb^2+^ than TSH, driven by the electron‐donating methoxy group and p‐π conjugation of the benzothiazole ring. These findings establish AMBT as the superior passivator for defect suppression in CsPbI_3_ perovskite films. To confirm that neither additive promotes 2D perovskite phase formation, solution‐state ^1^H NMR spectroscopy was performed by comparing pure TSH and AMBT with their respective PbI_2_ mixtures in DMSO‐d_6_ (Figure S3), revealing only minimal chemical shift changes consistent with surface coordination rather than lattice intercalation as a 2D spacer cation. The persistence of both additives in the annealed films, evidenced by the retained XPS chemical shifts of N 1s (412.94 → 413.67/413.69 eV) and O 1s (545.5 → 545.8 eV) after 180°C annealing (Figure 2a,b and Figure S2), confirms that the coordination bonds between the additives and Pb^2+^ are thermally stable and that both TSH and AMBT remain at grain boundaries and film surfaces in the final device, where they continue to passivate defects and suppress nonradiative recombination.

**FIGURE 3:**
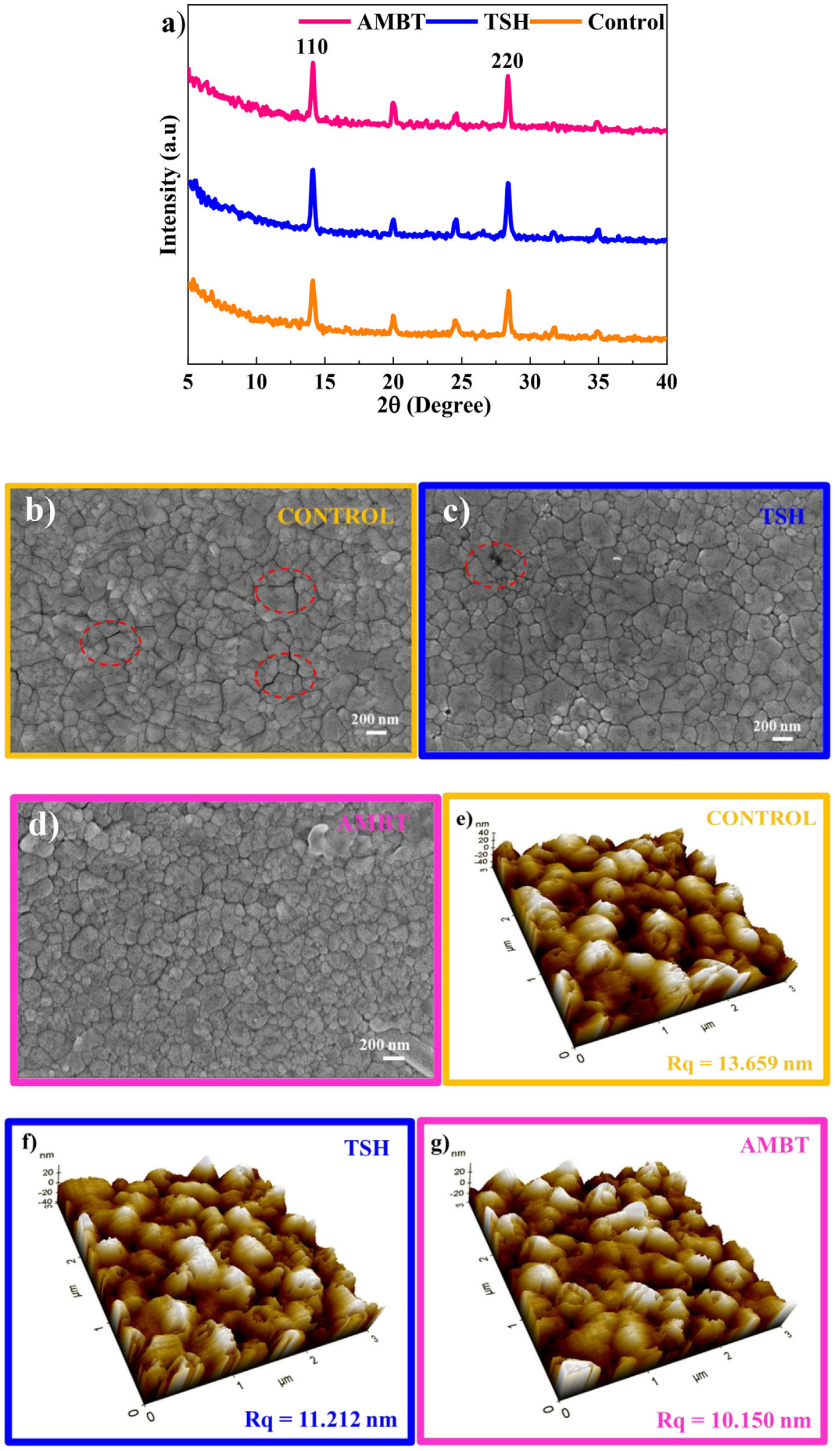
(a) XRD patterns of the control and additive modified CsPbI_3_ films. (b–d) SEM images. (e–g) AFM images of the control and additive modified CsPbI_3_ films.

To evaluate the influence of additive molecules on the crystallinity and structural evolution of CsPbI_3_ perovskite films, X‐ray diffraction (XRD) analysis was performed. As shown in (Figure 3a), all samples exhibit characteristic perovskite diffraction peaks at 2θ = 14.1° and 28.4°, corresponding to the (110) and (220) crystallographic planes, respectively. Crystallite sizes estimated via the Debye‐Scherrer equation reveal that the AMBT‐modified film possesses the largest average crystallite size of 33.69 nm, surpassing those of the TSH‐modified (30.15 nm) and control (28.96 nm) films, confirming that AMBT incorporation promotes superior crystal growth and enhanced crystallinity. Microstrain analysis of the (110) and (220) diffraction peaks (Table S4) further demonstrates that the control film exhibits the highest lattice strain, whereas AMBT modification effectively reduces microstrain. The elevated microstrain is known to trigger phase transitions and induce structural distortions in CsPbI_3_, its suppression by AMBT signifies improved structural stability of the perovskite lattice, which is essential for maintaining the photoactive phase under operating conditions. Low‐angle XRD measurements (2θ = 5°‐10°) show no evidence of diffraction peaks associated with layered 2D perovskite structures in either TSH‐ or AMBT‐modified films, indicating that no 2D phase formation occurs. To further assess the effect of the additives on grain structure and surface morphology, top‐view SEM and AFM measurements were conducted. As shown in (Figure 3b and Figure S4a), the control film displays a highly nonuniform surface characterized by numerous pinholes and small grain domains. In contrast, the AMBT‐modified film exhibits a markedly improved morphology, featuring enlarged grains, a smoother surface, and complete elimination of pinholes, collectively indicating a substantial reduction in defect‐rich regions (Figure 3d and Figure S4c). AFM analysis corroborates these observations, showing that the AMBT‐treated film achieves the lowest root‐mean‐square roughness (Rq = 10.15 nm) relative to 11.21 nm for TSH and 13.66 nm for the control film (Figure 3e–g). The improved surface smoothness and interfacial quality are expected to facilitate more efficient charge transport and suppress interfacial recombination, thereby benefiting overall device performance.

The observed morphological enhancements can be attributed to additive‐induced modulation of crystallization kinetics. FTIR and XPS analyses suggest that both TSH and AMBT interact with undercoordinated Pb^2+^ ions during film formation, decreasing the availability of free Pb^2+^ for rapid nucleation. This suppression of fast nucleation mitigates the formation of small grains and high defect densities typically observed in pristine CsPbI_3_ films, favoring crystal growth and leading to larger, more uniform grains. Consequently, the average grain size increases from 148.69 nm in the control film to 217.36 and 228.17 nm for TSH‐ and AMBT‐treated films, respectively (Figure S4). Notably, the bidentate coordination of AMBT with Pb^2+^ is stronger than the monodentate interaction of TSH, resulting in a more stable coordination environment in solution. This accounts for the larger grain size, reduced surface roughness, and superior film quality observed in AMBT‐treated films. Moreover, the absence of pinholes in these films indicates that bidentate coordination more effectively regulates crystallization, suppressing defect formation at grain boundaries and yielding high‐quality films suitable for efficient PSC applications.

**FIGURE 4:**
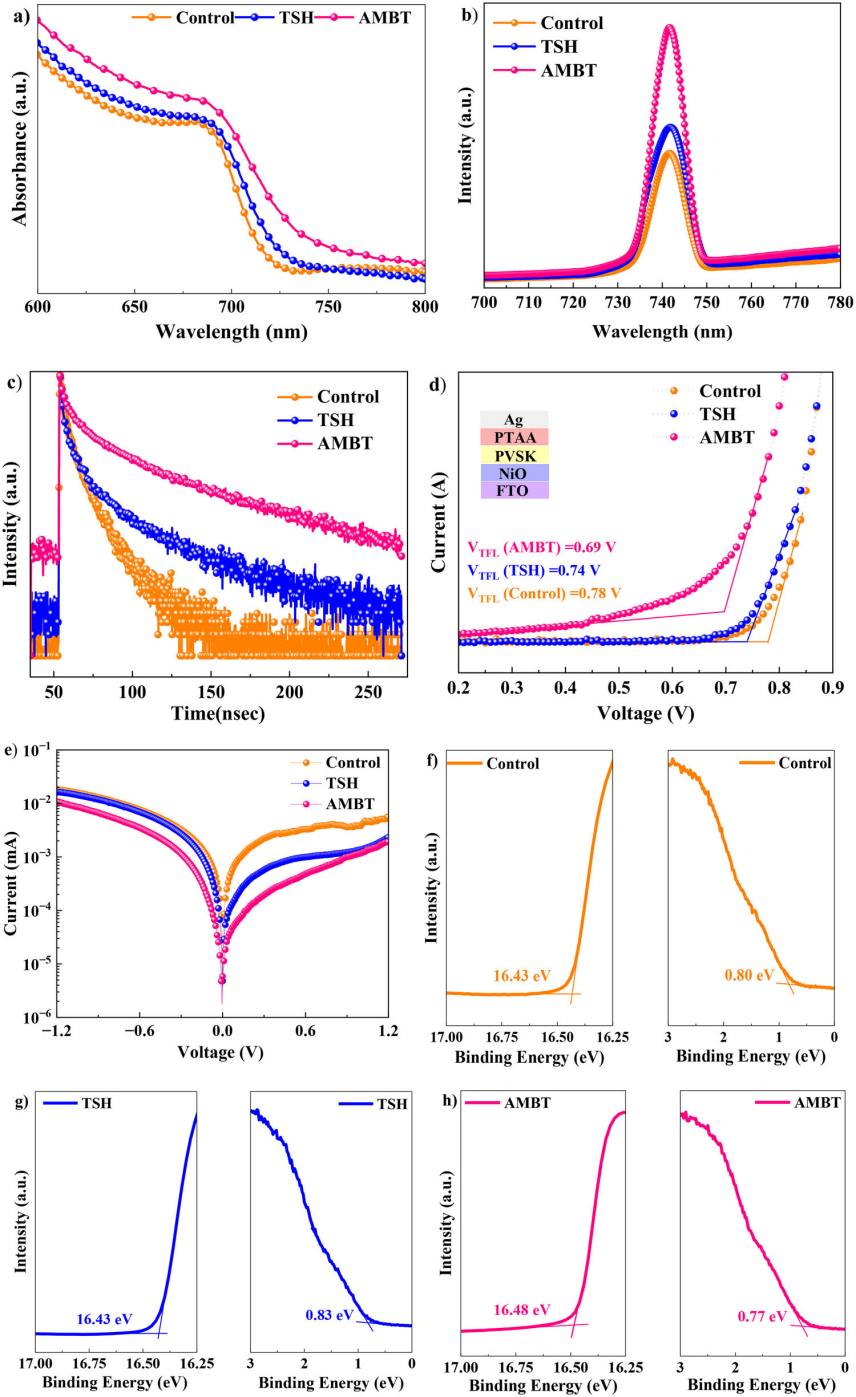
(a) UV–vis absorption spectra. (b) The steady‐state PL spectra. (c) TRPL spectra for the control and additive modified CsPbI_3_ films (d) Hole‐only devices with a structure of FTO/NiO_x_/perovskite/PTAA/Ag. (e) Dark *I*–*V* curves. (f–h) UPS spectra of the control and additive modified CsPbI_3_ films.

UV–vis absorption spectra reveal that the incorporation of TSH and AMBT induces a slight but progressive redshift in the absorption onset relative to the control film (Figure 4a), which is corroborated by Tauc plot analysis yielding optical bandgaps of 1.715 and 1.70 eV for TSH‐ and AMBT‐modified films, respectively, compared to 1.73 eV for the control (Figure S5). This modest bandgap narrowing suggests that the additives promote improved crystal quality or phase stabilization without disrupting the β‐phase formation of CsPbI_3_.Steady‐state PL study demonstrates that AMBT significantly diminishes nonradiative recombination, giving a ∼3.67‐fold enhancement of emission intensity (Figure 4b) [54, 55]. Finally, the TRPL study discloses the prolonged carrier lifetime from 5.65 to 31.33 ns (Figure 4c and Figure S6 and Table S2) [56]. The results are supported by SCLC measurements, which further indicate that trap‐filled limit voltages V_TFL_ decrease in both hole‐ and electron‐only devices, resulting in reduced trap densities Nt from 2.87 × 10^15^ to 2.54 × 10^15^ cm^−3^ and from 5.23 × 10^14^ to 4.38 × 10^14^ cm^−3^, respectively (Figure 4d and Figure S7 and Table S3) [54]. Accordingly, dark current density has been minimized, which directly demonstrates that the use of AMBT effectively passivates the defects and enhances the electronic quality of CsPbI_3_ films. The energy‐level alignment of perovskite films with and without additive treatment was investigated using ultraviolet photoelectron spectroscopy (UPS) (Figure 4f–h and Table S5), (Figure S8) displays a comparable device energy diagram. With conduction and valence band edges at 3.88 and 5.57 eV, respectively, the control film displays a Fermi level of 4.77 eV. The AMBT‐modified film, on the other hand, exhibits upshifted band edges (CBM: 3.80 eV; VBM: 5.49 eV) and a shallower Fermi level (4.72 eV). The interfacial energy alignment with the charge‐transport layers is enhanced by this band‐edge modulation. While the increased CBM promotes electron transfer to C_60_, the decreased offset between the perovskite VBM and the PTAA hole‐transport layer (5.24 eV) preserves a suitable driving force (0.25 eV) for effective hole extraction. As a result, there is an increase in quasi‐Fermi level splitting, improved fill factor (FF) and open‐circuit voltage (V_OC_), and improved charge separation and transport [53].

**FIGURE 5:**
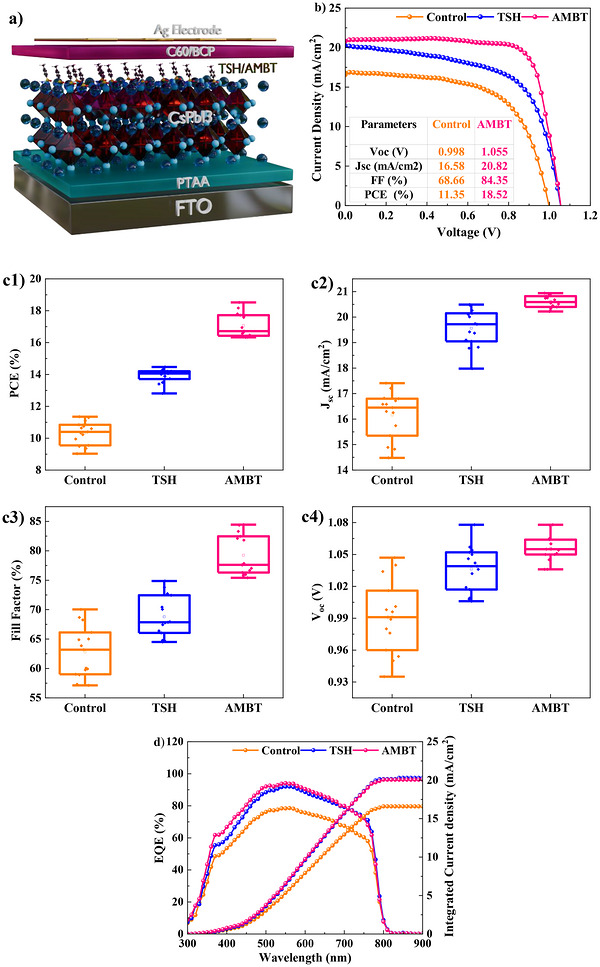
(a) Schematic of the FTO/PTAA/CsPbI_3–_TSH (or) AMBT/C_60_/BCP/Ag device architecture. (b) *J*–*V* characteristics of the corresponding PSCs. C1‐4) Statistical distributions of photovoltaic parameters. (d) External quantum efficiency (EQE) spectra.

To assess the effect of AMBT additives on device performance, inverted PSCs with the structure were fabricated (Figure 5a). The champion AMBT‐treated device (active area: 0.04 cm^2^) showed significantly improved photovoltaic performance: V_OC_ of 1.05 V, J_SC_ of 20.82 mA cm^−2^, an FF of 84.35%, and a PCE of 18.52%, outperforming the control devices (Figure 5b and Table 1). Statistical distributions from 15 devices per group (Figure 5c) confirm improved reproducibility and systematically higher V_OC_, consistent with improved interfacial energetics. EQE spectra gave integrated photocurrents of 16.20 and 20.43 mA cm^−2^ for the control and AMBT‐treated devices, respectively, in excellent agreement with *J*–*V* data (Figure 5d). Under 80% RH, AMBT‐treated devices outperformed the recent β‐CsPbI_3_ PSCs (Table S6).

**TABLE 1:** Device parameters of CsPbI_3_ PSCs with and without additives.

Samples	V_OC_ (V)	J_SC_ (mA·cm^−2^)	FF (%)	PCE (%)
Control	0.99	16.58	68.66	11.35
w TSH	1.05	20.26	67.76	14.47
w AMBT	1.05	20.82	84.35	18.52

A concentration of 2.0 mg mL^−1^ was found to be the optimal surface treatment for TSH (Figures S9 and S10 and Table S7) and 4.0 mg mL^−1^ for AMBT (Figures S11 and S12; Table S8). Stability tests were performed on both films and devices. Upon accelerated humidity exposure (80% RH; Supplementary time‐lapse Video S1, AMBT‐modified CsPbI_3_ film is represented as number 1, Control as number 2 and TSH‐modified CsPbI_3_ film as number 3 in the video), the control CsPbI_3_ film formed the non‐perovskite δ‐phase within 7 min and fully degraded by 20 min. Accordingly, TSH‐treated films underwent delayed degradation at ∼15 min, while the AMBT‐modified films resisted phase transition upon humidity for ∼40 min, which is the longest phase transition stability ever reported for CsPbI_3_ so far. This enhanced phase stability is linked to the enhanced crystallinity, reduced surface roughness, and higher water contact angle in the films treated with AMBT (Figure S13).

**FIGURE 6:**
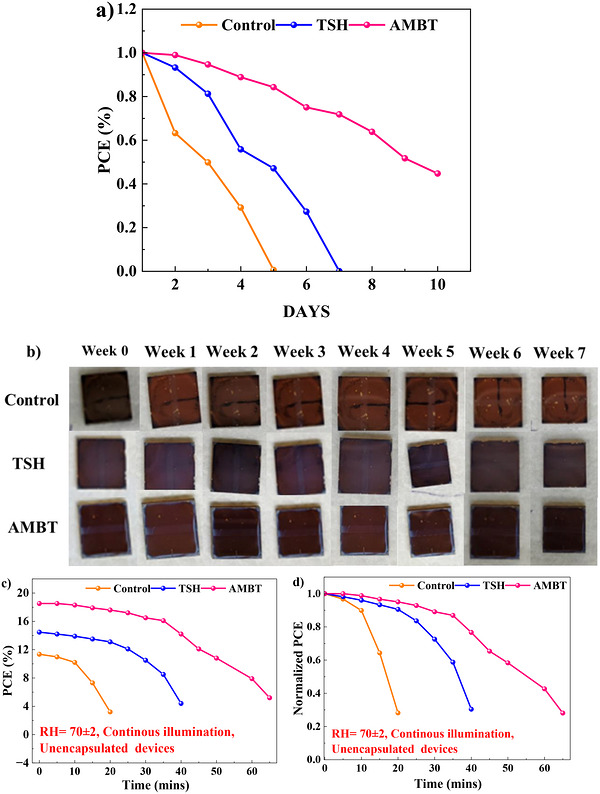
(a) Normalized photovoltaic efficiency of AMBT/TSH compositions and control. (b) Photographs of control and additive‐modified CsPbI_3_ films aged under ambient conditions (20% RH). Illumination stability of unencapsulated control, TSH‐modified, and AMBT‐modified CsPbI_3_ PSC under continuous 1‐sun AM1.5G illumination at RH = 70 ± 2%. (c) Absolute PCE versus time. (d) Normalized PCE versus time.

Open‐air stability tests over 10 days were performed for unencapsulated devices at ∼80% RH. The control films rapidly converted to the δ‐CsPbI_3_ phase within ∼15 min, while AMBT‐treated films delayed this transition to ∼80 min (Supplementary time‐lapse Video S1). Devices were thus measured immediately after fabrication and stored at 20% RH between measurements. The control devices failed within 3 days, and the TSH‐treated devices within 5 days, whereas the photoactive phase in the AMBT‐modified devices was preserved with only minor surface changes after 7 days. Figure 6a and Figure S14 show that, after 10 days, AMBT‐treated PSCs retained ∼40% of their initial efficiency compared to the fully degraded control and TSH devices. CsPbI_3_ and additive modified CsPbI_3_ thin films showed negligible morphological changes when stored at 20% RH, even after 8 weeks, as shown in Figure 6b and Figure S15. The improved environmental stability results from reduced defect density combined with enhanced crystallinity that limits moisture ingress and suppresses the δ‐phase transition.

To further evaluate the operational stability of the fabricated devices, illumination stability tests were performed on unencapsulated control, TSH‐modified, and AMBT‐modified devices under continuous 1‐sun AM1.5G illumination at RH = 70 ± 2% (Figure 5c,d). The device performance was measured every 5 mins. The control devices exhibited rapid PCE degradation under simultaneous light and humidity exposure, retaining only ∼30% of their initial efficiency within approximately 20 min. The TSH‐modified devices showed improved but insufficient stability, reaching the same 30% retention threshold at ∼40 min. In stark contrast, the AMBT‐modified devices maintained over 85% of their initial PCE during the first 25 min of continuous illumination and retained ∼30% of initial efficiency after 65 min, representing a 3.25‐fold improvement over the control. This hierarchy of illumination stability (AMBT > TSH > Control) is fully consistent with the defect passivation quality established by our DFT, XPS, FTIR, TRPL, and SCLC measurements. The stronger bidentate coordination of AMBT with surface Pb^2+^ more effectively suppresses light‐induced ion migration and photoinduced defect formation at grain boundaries, thereby providing robust protection against the combined light and humidity stress that unencapsulated CsPbI_3_ devices typically encounter under operational conditions.

## 3 Conclusions

In summary, we have shown that molecular passivation using 2‐amino‐6‐methoxybenzothiazole (AMBT) offers a very effective approach to stabilize CsPbI_3_ perovskite solar cells (PSC) against extremely humid conditions. Compared with monodentate TSH, the bidentate coordination of AMBT significantly increases its interaction energy with the perovskite surface, which in turn reduces defect density, enhances crystallinity, and greatly reduces nonradiative recombination. Such improvements allow the photoactive β‐phase in AMBT‐modified CsPbI_3_ films to persist for over 60 min at 80% RH well beyond that of both control and TSH‐treated films. As a result, unencapsulated devices based on AMBT reach a PCE of 18.52% and retain 40% of their initial efficiency after 10 days at 80% RH, constituting major progress in humidity‐resistant CsPbI_3_ PSCs. This work emphasizes the importance of strong bidentate molecular interactions to achieve long‐term environmental stability and offers a promising route toward practical deployment of all‐inorganic perovskite photovoltaics.

## Author Contributions

K.E. performed in software, resources, formal analysis, writing, review, and editing. T.V. performed in original draft preparation, review and editing, formal analysis, and data curation. L.‐C.C. performed in conceptualization, methodology, research oversight, validation, resources, project management, obtaining funds, formal analysis, review, and editing.

## Conflicts of Interest

The authors declare no conflicts of interest.

## Supporting information




**Supporting File 1**: smll73854‐sup‐0001‐SuppMat.docx.

**Supporting File 2**: smll73854‐sup‐0002‐VideoS1.mp4.

## Data Availability

The data that support the findings of this study are available in the supplementary material of this article.
