# Supplementary material for: Enhancing High‐Humidity Stability of CsPbI3 Perovskite Solar Cells Through Strong Bidentate Ligand Coordination
Source: Small. 2026 May 20;22(38):e73854. doi: 10.1002/smll.73854 (PMC13351536; doi:10.1002/smll.73854)
Supplement: Supplementary file 1 — Supporting File 1: smll73854‐sup‐0001‐SuppMat.docx. [file SMLL-22-e73854-s002.docx]

**Supporting Information**

**Enhancing High-Humidity Stability of CsPbI_3_ Perovskite Solar Cells through Strong Bidentate Ligand Coordination**

Karthikeyan Embrose, Thangaraji Vasudevan and Lung-Chien Chen^*^

National Taipei University of Technology, Taipei 10608, Taiwan;

karthikeyanembrose@gmail.com (K. E); orgthangaraji@gmail.com (T. V);

----------- ꝏ -----------

* **Corresponding Author:** ocean@ntut.edu.tw; Tel.: +886-2-2771-2171.

**Outline**

1. Experiment Materials

2. Fabrication of Perovskite Solar Cell

3. Computational Methods

4. Film Characterization

5. Device Characterization

6. Binding Energy Calculation

7. TRPL analysis

8. SCLC measurement

9. XRD measurement

10. Experimental figures and tables

**1. Experiment Materials**

Dimethylformamide (DMF, anhydrous, 99.8%), dimethyl sulfoxide (DMSO), 2‐propanol (IPA, anhydrous, 99.9%), chlorobenzene (CB, anhydrous,99.8%), and ethanol (EtOH, ACS/HPLC, 99.9%) were purchased from J&K Scientific. Cesium iodide (CsI, 99.999%) and Lead iodide (PbI_2_, 99.99%) were obtained from Sigma‐Aldrich, PTAA (> 98.0%) was obtained from Luminescence technology corp. Ltd, p-toluenesulfonyl hydrazide (TSH, 98%), and 2-amino-6-methoxybenzothiazole (AMBT) (AMBT, 98%) were bought from TCI. C_60_ was purchased from Nano‐C., dimethylamine hydroiodide (DMAI, 99.5%), and BCP (> 99%) were sourced from Xi'an Yuri Solar Co. Ltd. All materials were used as received without further treatment.

**2. Fabrication of Perovskite Solar Cell**

The patterned FTO glass substrates were sequentially cleaned with detergent, deionized water, acetone, and isopropyl alcohol via ultrasonication, then dried in an oven at 100°C. After complete drying, the substrates were subjected to UV‐ ozone treatment for 20 min. Next, the PTAA (2 mg·mL^–1^ in CB) was spin‐coated onto the substrates at 3000 rpm for 30 s and annealed at 100°C for 10 min. The films were then transferred into the glove box. For perovskite layer deposition, a precursor solution containing DMAI: CsI: PbI_2_ (0.8 M, molar ratio = 1.2:1:1) with various additives (2 mg·mL^–1^ TSH and 4 mg·mL^–1^ AMBT) was then spin‐coated onto the hole transport layer substrates: first at 1000 rpm for 10 s, followed by 2000 rpm for 30 s, followed by annealing at 180°C for 8 min under controlled ambient conditions (~20% relative humidity). Finally, C_60_ (20 nm), BCP (5 nm), and Ag (100 nm) were sequentially evaporated by a vacuum thermal coater (< 5×10^–6^ Pa) with evaporation rates of 0.5, 0.5, and 1 Å·s^-1^, respectively.

**3. Computational Methods**

The theoretical calculations were performed via the Gaussian 16 suite of programs. The structures of the studied molecules (AMBT & TSH) were fully optimized at the B3LYP-D3BJ/TZVP level of theory. The structures were characterized as a local energy minimum on the potential energy surface by verifying that all the vibrational frequencies were real. The Gaussian 09W program was used to plot the color-filled iso-surface graphs to visualize the molecular electrostatic potential (ESP).

Density functional theory (DFT) calculations were performed using the Quantum ESPRESSO package within the generalized gradient approximation (GGA) using the Perdew–Burke–Ernzerhof (PBE) functional. Electron–ion interactions were treated using PS Library PAW-type pseudopotentials for Cs, Pb, I, C, N, O, S, and H. A plane-wave basis set was employed with kinetic-energy cutoffs of 60 Ry for the wavefunctions and 480 Ry for the charge density. Electronic minimization was carried out using a fixed-occupations scheme with Gaussian smearing of 0.0275 Ry. Self-consistent field (SCF) iterations were converged to 2.4 × 10^-8^ Ry, and structural relaxations were considered converged when the residual forces were below 1.0 × 10^-3^ Ry Bohr^-1^. Dispersion interactions were included using the Grimme DFT-D3 correction when indicated.

The equilibrium lattice parameters of orthorhombic γ-CsPbI_3_ were optimized to a = 8.659 Å, b = 8.915 Å, c = 12.653 Å. These optimized bulk values ​​were then used to construct a γ-CsPbI_3_ (110) surface slab with p (1×1) periodicity. The slab contained one stoichiometric layer and was separated from its periodic images by a 15 Å vacuum layer along the surface normal. During structural optimization, the bottom half of the slab was fixed, whereas the top half was fully relaxed. Brillouin-zone sampling was carried out using a 2 × 2 × 1 Monkhorst–Pack grid.

**4. Film Characterization**

UV‐vis absorption spectra were acquired using a UV‐2600 spectrophotometer (Shimazu). Steady‐state PL spectra were recorded on an FLS1000 fluorescence spectrometer (Edinburgh). TRPL decay profiles were obtained with a Delta Flex time‐correlated single photon counting system (Horiba) under 375 nm pulsed laser excitation. RD patterns were collected on an Equinox 3500 diffractometer (Thermo Fisher) using Cu Kα radiation (λ = 1.5406 Å) at 40 kV, with 2θ scans from 2° to 90°. Field‐emission SEM images were acquired on a Sigma 300 microscope (Carl Zeiss). AFM measurements were performed under nitrogen using a Dimension Icon microscope (Bruker). XPS measurements were performed on an ESCALAB Xi+ X‐ray photoelectron spectrometer (Thermo Fisher) with binding energies referenced to C 1s at 284.8 eV. ¹H NMR spectrum recorded on a Bruker 600 MHz NMR spectrometer. The FTIR spectrum was measured by using Fourier Transform Infrared (VERTEX 70, Bruker, Germany). All measurements are carried out at room temperature.

**5. Device Characterization**

J-V curves of the perovskite solar cells characteristics were collected by a standard spectrum solar simulator (Xenon lamp) equipped with a Keithley 2400 source meter (Enlitech, SS‐X_AM1.5G). The light intensity of a single light source simulator was calibrated by a certified mono‐Si reference cell. EQE measurements were acquired by a solar cell quantum efficiency testing system (SCS600; Zolix), calibrated by a mono‐Si standard solar cell (QE‐B3; Zolix).

**6. Binding Energy Calculation:**

The following formula illustrates the mathematical technique for determining the adsorption energy using DFT energy:

$$\begin{aligned} E_{\mathrm{ads}}=E_{surface\_mol}-E_{\mathrm{surface}}-E_{\mathrm{mol}}\#\left( 1 \right) \end{aligned}$$

where E_surface_mol_ represents the energy that molecules have adsorbed onto the surface, E_surface_ represents the energy of the faulty surface, and E_mol_ represents the energy of the adsorption molecule the energy of the isolated adsorbate in a 20 Å cubic box sampled using a 1 × 1 × 1 k-point mesh.

**Table S1.** The calculated binding energy of the adsorbed TSH/AMBT molecules on the defective perovskite surface.

|  | **E_surface_mol_(eV)** | **E_surface_(eV)** | **E_mol_(eV)** | **E_ads_(eV)** |
| --- | --- | --- | --- | --- |
| TSH | -34617.63 | -31553.05 | -3063.60 | -0.98 |
| AMBT | -34316.97 | -31553.05 | -2762.01 | -1.91 |

**7.** **TRPL analysis:**

The time-resolved photoelectron spectra and TAS dynamic bleaching recovery were fitted to a tri-exponential decay function:[1]

$\begin{aligned} \text{A(t) = }\text{A}_{\text{1}}\exp\left( \text{-}\frac{\text{t}}{\text{τ}_{\text{1}}} \right)\text{ +}\text{ A}_{\text{2}}\exp\left( \text{-}\frac{\text{t}}{\text{τ}_{\text{2}}} \right)\text{ +}\text{ A}_{\text{3}}\exp\left( \text{-}\frac{\text{t}}{\text{τ}_{\text{3}}} \right)\#\left( 2 \right) \end{aligned}$

In the equation, A_1_, A_2_, and A_3_ are the respective weighting factors of each decay channel, τ_1_, τ_2_, and τ_3_ represent the first, second, and third-order decay, in which the first-order decay is divided into a fast component and the second and third-order decay are divided into a slow component. The average lifetime's $\text{τ}_{\text{avg.}}$ of CsPbI_3_ perovskite was calculated by the following function:

$\begin{aligned} \text{τ}_{\text{avg}}\text{ = }\frac{\text{A}_{\text{1}}\text{τ}_{\text{1}}^{\text{2}}\text{ }{\text{+ }\text{A}_{\text{2}}\text{τ}_{\text{2}}^{\text{2}}\text{ + A}}_{\text{3}}\text{τ}_{\text{3}}^{\text{2}}}{\text{A}_{\text{1}}\text{τ}_{\text{1}}\text{ + }\text{A}_{\text{2}}\text{τ}_{\text{2}}\text{ + A}_{\text{3}}\text{τ}_{\text{3}}}\#\left( 3 \right) \end{aligned}$

**Table S2**. TRPL lifetime parameters of the control and additive-modified CsPbI_3_ perovskites.

|  | **A_1_** | **τ_1_(ns)** | **A_2_** | **τ_2_(ns)** | **A_3_** | **τ_3_(ns)** | **τ_avg._ (ns)** |
| --- | --- | --- | --- | --- | --- | --- | --- |
| Control | 9291.16 | 1.04 | 3907.46 | 4.33 | 827.40 | 12.26 | 5.65 |
| w TSH | 4309.66 | 3.94 | 38864.63 | 0.76 | 658.29 | 22.68 | 6.95 |
| W AMBT | 9184.25 | 1.29 | 3743.37 | 8.06 | 1669.01 | 45.34 | 31.33 |

**8. SCLC measurement**:

The trap densities for electrons and holes were calculated utilizing the space-charge limited current (SCLC) method:[2, 3]

$$\begin{aligned} n_{t\left( e/h \right)}=\frac{2\varepsilon_{r}\varepsilon_{0}V_{\mathrm{TFL}\left( e/h \right)}}{eL^{2}}\#\left( 4 \right) \end{aligned}$$

where ε_0_ is the permittivity of vacuum, ε_r_ is the relative dielectric constant (ε_r_ = 3), e is the elementary charge of the electron, and *L* is the thickness of the perovskite films (L = 300 nm). V_TFL_(e/h) is the trap ﬁlling voltage.

**Table S3.** Trap filling voltage (V_TFL)_ and trap density (N_t_) values of the hole only and electron only devices.

| **Samples** | **Hole only device** | | **Electron only device** | |
| --- | --- | --- | --- | --- |
|  | **V_TFL_ (V)** | **N_t_ (cm^-3^)** | **V_TFL_ (V)** | **N_t_ (cm^-3^)** |
| Control | 0.78 | 2.87×10^15^ | 0.142 | 5.23×10^14^ |
| w TSH | 0.74 | 2.74×10^15^ | 0.125 | 4.60×10^14^ |
| W AMBT | 0.69 | 2.54×10^15^ | 0.119 | 4.38×10^14^ |

**9. XRD measurement:**

The Crystallite size and strain in the samples were calculated using the Debye Scherrer's equation [4-7].

$$\begin{aligned} D=\frac{k\lambda}{\beta cos\theta}\boldsymbol{\#}\left( \boldsymbol{5} \right) \end{aligned}$$

Where, K=Scherrer constant (0.94), λ=Wavelength of X-ray source (λ=1.5406 Å), θ=Bragg's angle & β=Full width at half maximum (FWHM) (taken from 110 and 220 peaks), D=Crystallite size. The Crystallite sizes obtained from the above equation are 4.88 nm and 5.22nm for the control, 5.20 nm and 5.31nm for TSH and 5.93 nm and 5.82nm for AMBT.

Furthermore, the microstrain (*ε*) were calculated from the XRD data using the following formula,

$$\begin{aligned} \varepsilon=\frac{\beta}{4tan\theta}\#\left( 6 \right) \end{aligned}$$

where *n* is a factor that is nearly equal to the minimum dislocation density. The calculated values of the microstrain and dislocation density.

The 110 and 220 peaks of the control sample exhibit the highest microstrain levels compared to TSH and AMBT samples. Such strain can induce phase transitions in crystal structures, leading to substantial alterations in the material's phase diagram. Conversely, a more stable crystal lattice typically corresponds to smaller microstrain.

**Table S4.** Summary of the Crystallite Sizes (D) and Microstrain (ε) obtained from XRD.

| **Parameters** | | **Diffraction**  **Peak** | **Peak Position**  **(2θ)** | **FWHM**  **β(θ)** | **D (nm) Kλ/βcosθ** | **Micro strain (ε)**  **β/4tanθ** |
| --- | --- | --- | --- | --- | --- | --- |
| Control | K = 0.94  𝜆 = 1.5406 Å | 110 | 14.125 | 0.29854 | 28.01 | 0.0105 |
|  |  | 220 | 28.375 | 0.28603 | 29.92 | 0.0049 |
| TSH | K = 0.94  𝜆 = 1.5406 Å | 110 | 14.125 | 0.28015 | 29.84 | 0.0098 |
|  |  | 220 | 28.375 | 0.28092 | 30.47 | 0.0048 |
| AMBT | K = 0.94  𝜆 = 1.5406 Å | 110 | 14.125 | 0.24584 | 34.01 | 0.0086 |
|  |  | 220 | 28.375 | 0.25631 | 33.40 | 0.0044 |

**10. Experimental figures and tables:**


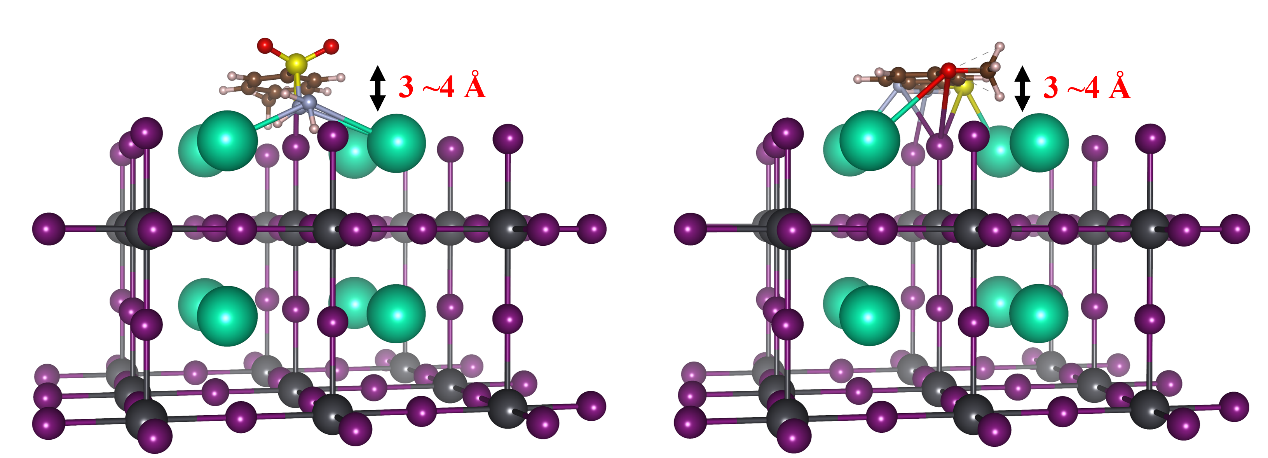


**b)**

**a)**

**Figure S1.** Theoretical model of coordinate bond formation between undercoordinated Pb^2+^/ Cs^+^ and a) TSH b) AMBT on CsPbI_3_ perovskite surface.

**Figure S2.** Perovskite film characterisation using XPS Control, TSH, and AMBT. a) N 1s b) O 1s c) Cs 3d.

^
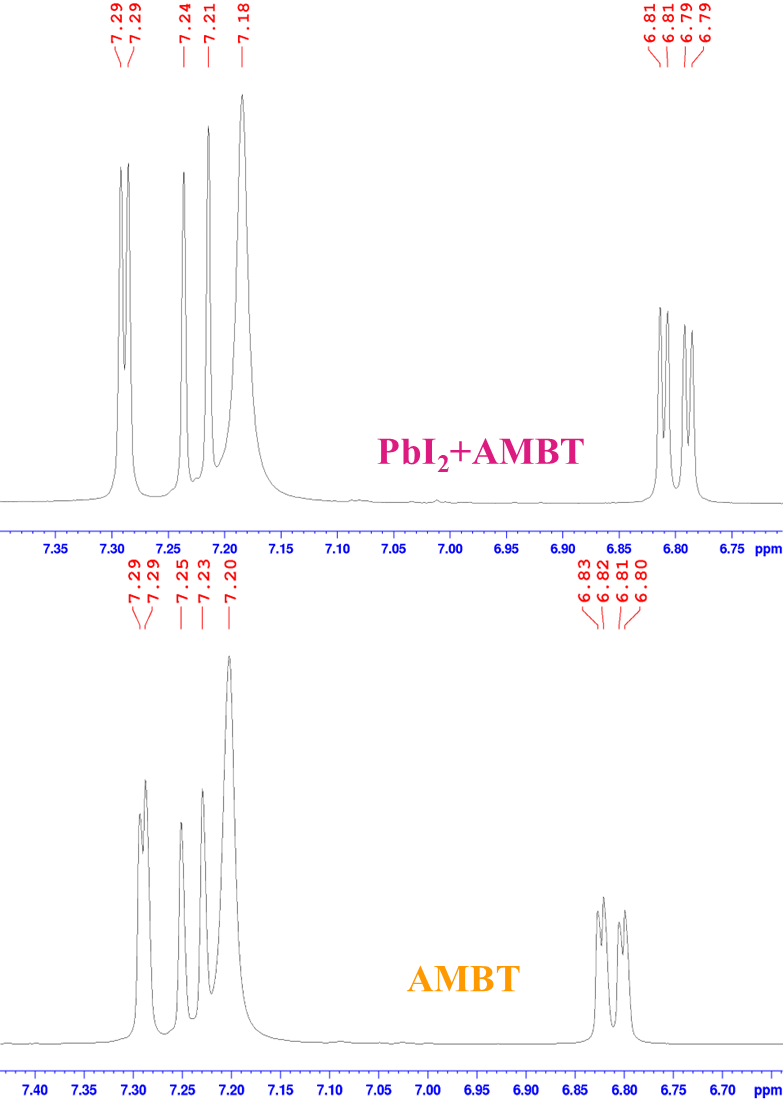
^

**a)**

^
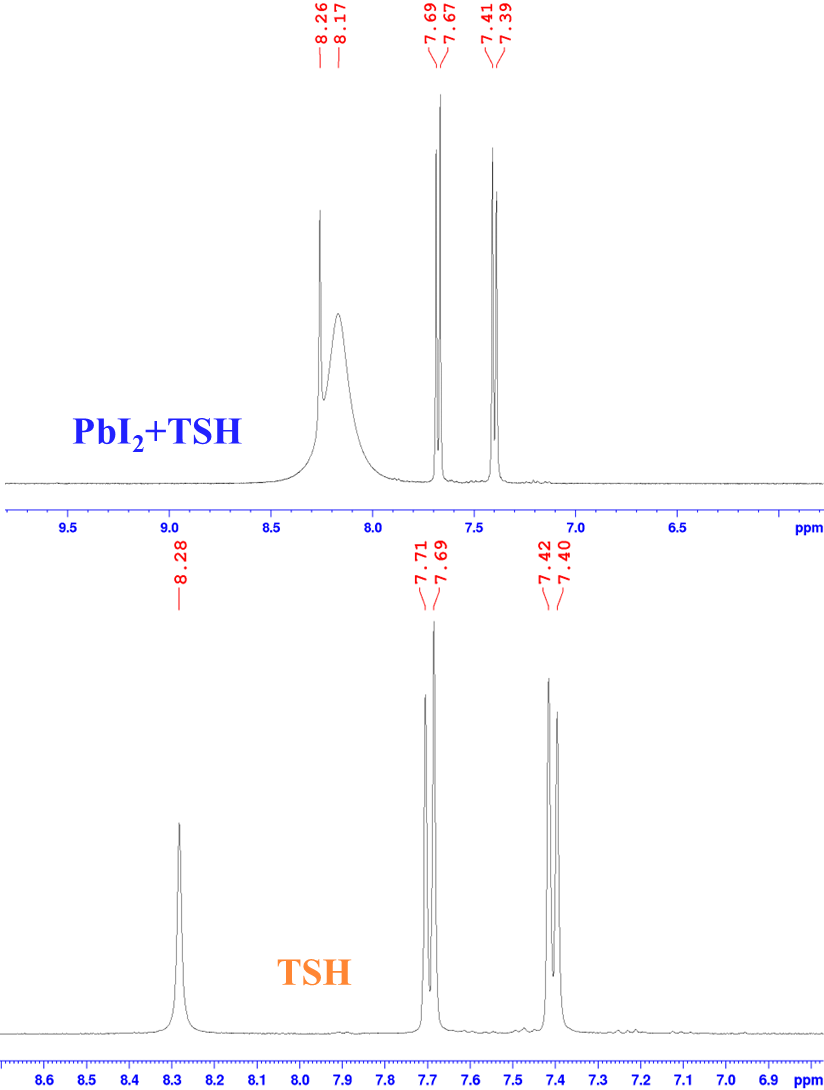
^

**b)**

**Figure S3.** Solution-state ¹H NMR spectra of (a) pure AMBT and AMBT-PbI_2_ mixture, and (b) pure TSH and TSH-PbI_2_ mixture in DMSO-d_6_.

To address whether the amine-containing additives TSH and AMBT could promote formation of 2D perovskite structures during thermal annealing, we performed solution-state ¹H NMR spectroscopy by comparing the spectra of pure TSH and AMBT with those of their respective PbI_2_ mixtures in DMSO-d_6_, which simulates the precursor solution environment (**Figure S3**). For AMBT (**Figure S3a**), the aromatic proton signals of the benzothiazole ring appear at δ = 7.18-7.29 ppm and δ = 6.79-6.81 ppm in the pure molecule. Upon mixing with PbI_2_, these signals shift by less than 0.04 ppm, and no new peaks emerge at chemical shifts inconsistent with free AMBT. Similarly, for TSH (**Figure S3b**), the para-substituted tolyl aromatic protons undergo only minor shifts of approximately 0.12 ppm upon PbI_2_ addition, with no appearance of new resonances. These observations are critically important for ruling out 2D perovskite formation. [8]

**Figure S4.** Histogram of grain size distribution for the control and additive-modified CsPbI_3_ perovskites.

**Figure S5.** Tauc plot of the films for the control and TSH-modified & AMBT-modified CsPbI_3_ perovskites.

**Figure S6.** TRPL spectra and its exponential fitting curve of the films for the control and TSH-modified & AMBT-modified CsPbI_3_ perovskites.

**Figure S7.** Electron-only devices with a structure of FTO/SnO_2_/perovskite/C_60_/Ag for the control and TSH-modified & AMBT-modified CsPbI_3_ perovskites.

**
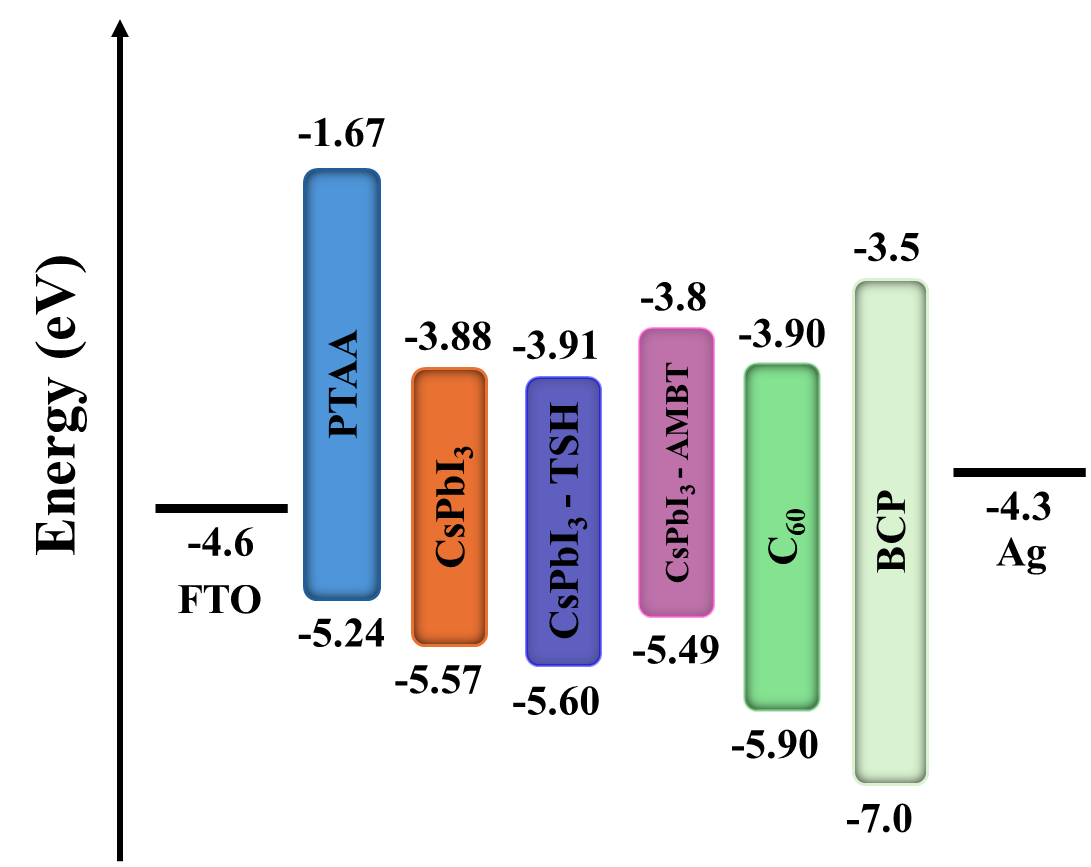
**

**Figure S8.** Energy level alignment diagram of the CsPbI_3_ and TSH-modified & AMBT-modified CsPbI_3_ perovskites**.**

**Table S5**. UPS evaluation of the optical characteristics of perovskite thin films with and without additive treatment.

|  | **E_cut-off_**  **(eV)** | **E_v_ - E_F_**  **(eV)** | **E_F_**  **(eV)** | **E_V_**  **(eV)** | **E_C_**  **(eV)** | **E_g_**  **(eV)** |
| --- | --- | --- | --- | --- | --- | --- |
| Control | 16.43 | 0.8 | 4.77 | 5.57 | 3.88 | 1.69 |
| w TSH | 16.43 | 0.83 | 4.77 | 5.6 | 3.91 | 1.69 |
| W AMBT | 16.48 | 0.77 | 4.72 | 5.49 | 3.8 | 1.69 |

**Table S6.** Summary of photovoltaic parameters for state-of-the-art inorganic PSCs fabricated under high humidity atmosphere.

| Perovskite | Humidity (RH) % | Voc (V) | FF  (%) | J_sc_  (mA/cm^2^) | PCE  (%) | Ref. |
| --- | --- | --- | --- | --- | --- | --- |
| CsPbI_3_ | 92 | 1.20 | 64 | 15.68 | 12.04 | [1] |
| CsPbI_2_Br | 91 | 1.27 | 78 | 15.20 | 15.11 | [2] |
| CsPbI_3_ | 60 | 1.09 | 78 | 18.31 | 15.91 | [3] |
| CsPb_0.8_Ge_0.2_I_2_Br | 55 | 1.27 | 70 | 12.15 | 10.80 | [4] |
| CsPbI_3_ | 60 | 1.09 | 84 | 17.92 | 17.68 | [5] |
| CsPbI_3_ | 60 | 1.16 | 79.4 | 20.45 | 18.39 | [6] |
| CsPbI_3_ | 80 | 1.16 | 79.3 | 19.70 | 18.18 | [7] |
| CsPbI_3_ | 80 | 1.05 | 84.35 | 20.82 | 18.52 | **This work** |

**Figure S9.** Statistical box charts of photovoltaic parameters as a function of devices

with TSH-modified CsPbI_3_ films with 0.04 cm^2^ active area: a) FF, b) V_OC_, c) J_sc_ and d) PCE. Data are extrapolated from the analysis of 15 solar cells.

**Figure S10.** J-V curve of the TSH‐modified device at different concentrations.

**Table S7.** Device parameters of TSH-modified CsPbI_3_ film with different concentrations.

| Samples | **V***_OC_* (V) | **J***_SC_* (mA·cm**^–^**^2^) | FF (%) | PCE (%) |
| --- | --- | --- | --- | --- |
| 1.0 mg/mL | 1.01 | 18.81 | 68.13 | 12.94 |
| 2.0 mg/mL | 1.05 | 20.26 | 67.76 | 14.47 |
| 4.0 mg/mL | 0.95 | 18.00 | 69.34 | 11.93 |

**Figure S11.** Statistical box charts of photovoltaic parameters as a function of devices

with AMBT-modified CsPbI_3_ films with 0.04 cm^2^ active area: a) FF, b) V_OC_, c) J_sc_ and d) PCE. Data are extrapolated from the analysis of 15 solar cells.

**Table S8.** Device parameters of AMBT-modified CsPbI_3_ film with different concentrations.

| Samples | **V***_OC_* (V) | **J***_SC_* (mA·cm**^–^**^2^) | FF (%) | PCE (%) |
| --- | --- | --- | --- | --- |
| 2.0 mg/mL | 0.98 | 19.40 | 66.10 | 12.59 |
| 4.0 mg/mL | 1.05 | 20.82 | 84.35 | 18.52 |
| 6.0 mg/mL | 1.05 | 20.73 | 72.72 | 15.94 |

**Figure S12.** J-V curve of the AMBT‐modified device at different concentrations.

**
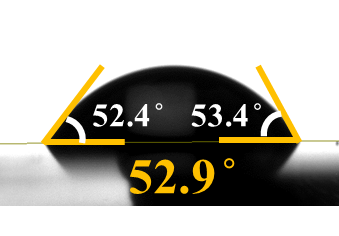
**

**b)**

**a)**


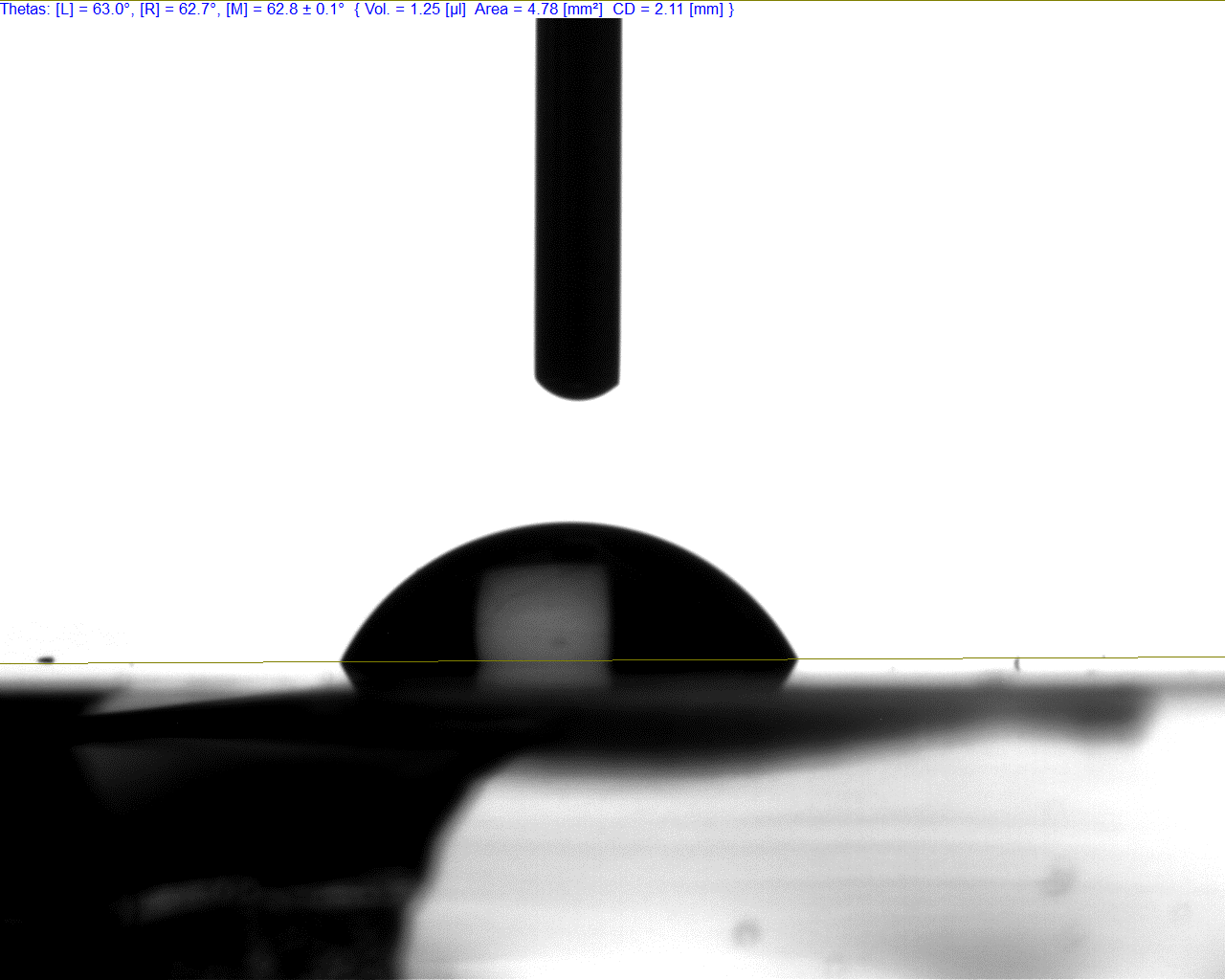


**62.8 ̊**

**63.0 ̊**

**62.7 ̊**

**c)**

**
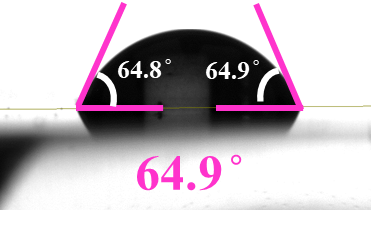
**

**Figure S13.** Water Contact angles for the a) control b) TSH-modified CsPbI_3_ c) AMBT-modified CsPbI_3_ perovskites.

**Figure S14.** PCE for the control, TSH and AMBT-modified CsPbI_3_ perovskites under RH 80%.

**
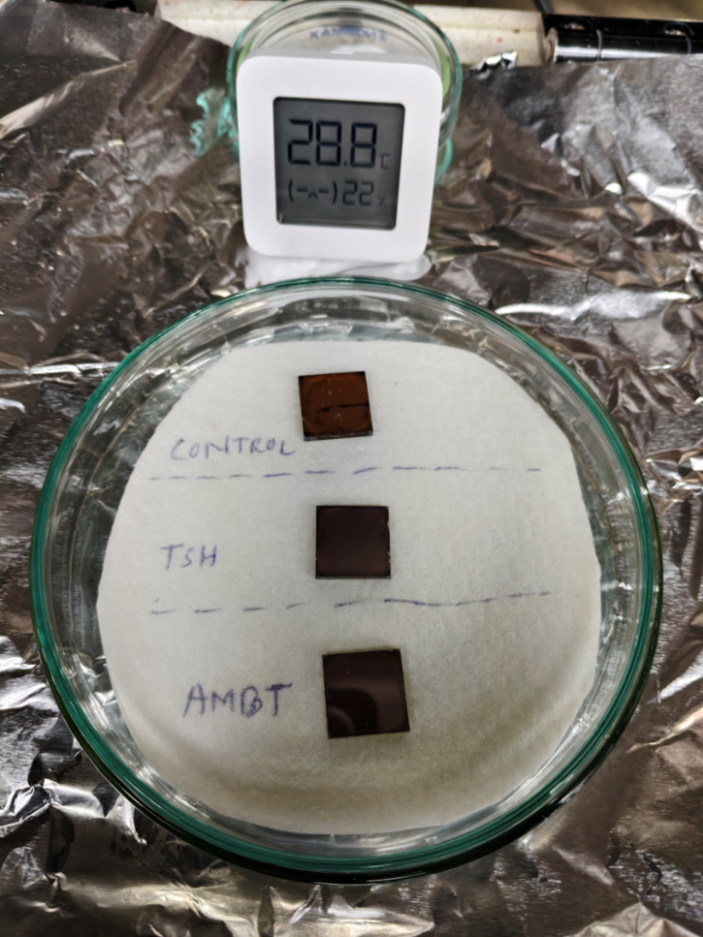
**

**Figure S15.** Photographs of control and additive-modified CsPbI_3_ films stored under ambient conditions (20% RH).

**References:**

1. Liang, J.; Wang, C.; Wang, Y.; Xu, Z.; Lu, Z.; Ma, Y.; Zhu, H.; Hu, Y.; Xiao, C.; Yi, X.; Zhu, G.; Lv, H.; Ma, L.; Chen, T.; Tie, Z.; Jin, Z.; Liu, J. Adv. Mater. **2019**, *31*, 1903448.
2. Duan, C.; Zhang, K.; Peng, Z.; Li, S.; Zou, F.; Wang, F.; Li, J.; Zhang, Z.; Chen, C.; Zhu, Q.; Qiu, J.; Lu, X.; Li, N.; Ding, L.; Brabec, CJ; Gao, F.; Yan, K. Adv. Energy Mater. **2020**, *10*, 2000691.
3. Liang, L.; Guo, Q.; Xue, Y.; Guo, X.; Lin, Y.; Chen, Y.; Zhang, W.; Liu, H.; Fang, JJ Mater. Chem. A **2019**, *7*, 26776–26784.
4. Yang, F.; Guo, Q.; Chen, Y.; Zhang, W.; Liu, H.; Fang, J. Angew. Chem. Int. Ed. **2018**, *57*, 12785–12789.
5. Lee, S.; Kim, D.; Park, H.; Jeong, J.; Song, H.; Kang, S. Commun. Mater. **2025**, *6*, 72.
6. Fu, S.; Li, X.; Wan, J.; Zhang, W.; Song, W.; Fang, J. Adv. Funct. Mater. **2022**, *32*, 2111116.
7. Lu, C.; Li, X.; Guo, X.; Fu, S.; Zhang, W.; Yuan, H.; Fang, J. Chem. Eng. J. **2023**, *452*, 139495.
8. Kim, H., Kim, J.S., Heo, J.M., Pei, M., Park, I.H., Liu, Z., Yun, H.J., Park, M.H., Jeong, S.H., Kim, Y.H. and Park, J.W., **Nat. Commun.** 2020, **11**, 3378.
